# Supplementary material for: Lexical selection with competing distractors: Evidence from left temporal lobe lesions
Source: Psychon Bull Rev. 2017 May 8;25(2):710–7. doi: 10.3758/s13423-017-1301-0 (PMC5902514; doi:10.3758/s13423-017-1301-0)
Supplement: Supplementary file 1 — (DOCX 87 kb) [file 13423_2017_1301_MOESM1_ESM.docx]

**Supplemental material**

**Materials**

The pictures used in the experiment, as well as name agreement norms (i.e., proportion of participants who gave the target name relative to the number of participants who named the picture) from the BOSS database (Brodeur et al., 2010) are shown in Table S1.

**Table S1.** Pictures used in the experiment and their name agreement from the BOSS database and from the present experiment.

| Picture | Target name in exp | Name agreement from BOSS (%) | Used for analyses | Name agreement from controls' first responses (%) | Remark |
| --- | --- | --- | --- | --- | --- |
| pants | pants | 79 | no | 100 | excluded because "shorts" was consistently named "pants" |
| scarf | scarf | 95 | yes | 100 |  |
| shirt01 | shirt | 44 | no | 61.5 | other names: jacket, coat |
| shorts01 | shorts | 74 | no | 30.8 | consistently named "pants" |
| drill01b | drill | 47 | yes | 100 |  |
| hammer01 | hammer | 100 | yes | 100 |  |
| pliers02b | pliers | 85 | yes | 100 |  |
| saw02b | saw | 88 | yes | 100 |  |
| cow | cow | 93 | yes | 100 |  |
| horse | horse | 100 | yes | 100 |  |
| pig | pig | 90 | yes | 100 |  |
| sheep | sheep | 93 | yes | 100 |  |
| cannon | cannon | 93 | yes | 100 |  |
| revolver | gun | 43 | yes | 100 |  |
| spear02 | spear | 75 | no | 23.1 | other names: arrow, paintbrush |
| sword01 | sword | 81 | yes | 100 |  |
| bowl01 | bowl | 67 | yes | 100 |  |
| jug | jug | 31 | no | 23.1 | consistently named "pitcher" |
| mug05 | mug | 50 | no | 30.8 | consistently named "cup" |
| plate01b | plate | 92 | yes | 92.3 |  |
| arm | arm | 71 | yes | 100 |  |
| ear | ear | 83 | yes | 100 |  |
| leg | leg | 60 | yes | 92.3 |  |
| nose | nose | 88 | yes | 100 |  |
| bed | bed | 81 | yes | 100 |  |
| chair | chair | 88 | yes | 100 |  |
| dresser02 | dresser | 49 | no | 61.5 | other names: bureau, chest, table |
| table01 | table | 81 | yes | 100 |  |
| apple07 | apple | 95 | yes | 100 |  |
| banana01 | banana | 100 | yes | 100 |  |
| lemon02 | lemon | 100 | yes | 100 |  |
| pear01 | pear | 100 | yes | 100 |  |
| acousticguitar02 | guitar | 71 | yes | 100 |  |
| drum01 | drum | 51 | yes | 92.3 |  |
| grandpiano | piano | 55 | yes | 100 |  |
| violin | violin | 97 | yes | 100 |  |
| barn | barn | 71 | no | 61.5 | other names: shed, house |
| castle | castle | 61 | yes | 69.2 |  |
| church | church | 81 | yes | 92.3 |  |
| shed02 | shed | 60 | no | 38.5 | other name: garage |
| binder03b | binder | 68 | no | 30.8 | other names: book, notebook |
| book01b | book | 90 | yes | 100 |  |
| pen04b | pen | 90 | yes | 100 |  |
| pencil01 | pencil | 97 | yes | 100 |  |
| airplane* | airplane | NA | yes | 100 |  |
| bicycle | bicycle | 43 | yes | 100 |  |
| bus | bus | 57 | yes | 100 |  |
| car | car | 81 | yes | 100 |  |
| bracelet01 | bracelet | 62 | no | 69.2 | other name: necklace, excluded because "necklace" was another picture in the experiment |
| necklace | necklace | 81 | no | 61.5 | other name: bracelet |
| ring01 | ring | 100 | no | 61.5 | other name: bracelet |
| watch02a | watch | 87 | no | 61.5 | other name: bracelet |
| broccoli01a | broccoli | 97 | yes | 100 |  |
| cucumber | cucumber | 95 | yes | 100 |  |
| lettuce | lettuce | 68 | yes | 92.3 |  |
| pumpkin | Pumpkin | 98 | yes | 100 |  |

* The picture “airplane” was taken from our own database. Exp = experiment

We calculated the proportion of control participants who named a given picture with its target name at its first presentation, that is, the first time participants encounter each picture in the experiment. The distribution of name-agreement values is shown in Figure S1 for the pictures included in the analyses and the pictures not included in the analyses. The actual name-agreement values are shown in Table S1 above. As can be seen, all but one item kept for analysis had name-agreement values above 90%. Two pictures had name agreement above 70% (“pants” and “bracelet”), but they were not kept in the analyses because other two pictures (“shorts” and “necklace”) were often named “pants” and “bracelet”.


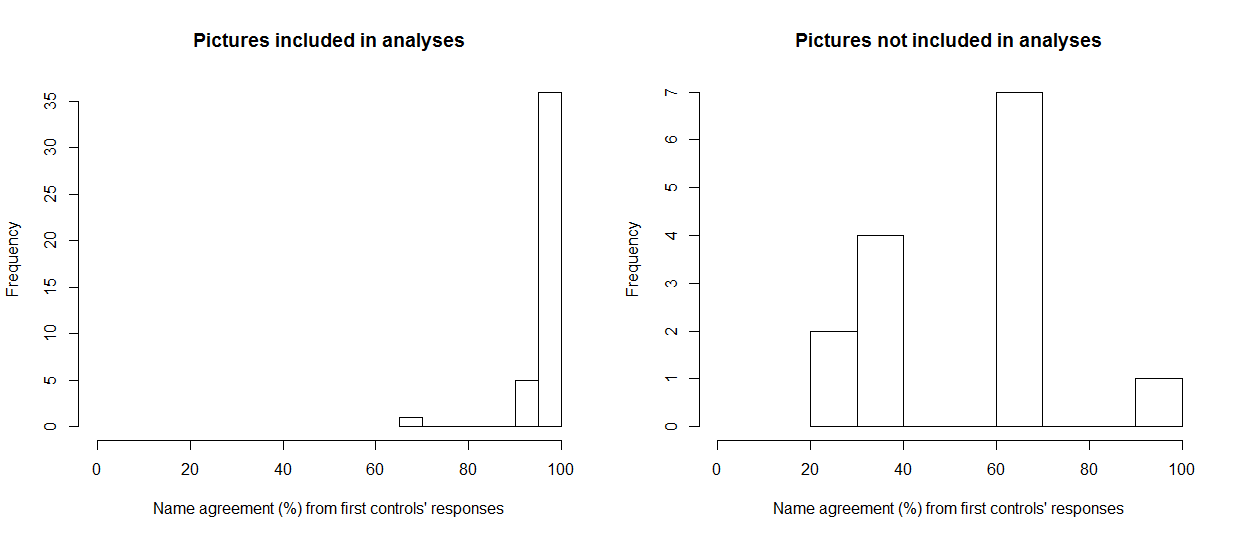


**Figure S1.** Distribution of name-agreement values, calculated from the first presentation of each item in the experiment for the control participants, for the picture items included in the analyses (left) and for those excluded from analyses (right).

We also ran the same analyses on the RTs as reported in the main text after excluding trials in an item-wise fashion. That is, if a participant named, for example, the picture item “lemon” incorrectly once, all other trials corresponding to the picture item “lemon” were also excluded for this participant, even if the other responses were correct.

Overall incongruency (unrelated vs congruent) and semantic interference (related vs unrelated) effects were observed (*p*s < .001). Patients were slower than controls (*p*s < .001). The incongruency effect was statistically larger for frontal and temporal patients than for controls (*p*s < .001). The semantic interference effect was significant for the controls (*p* < .001) and temporal patients (*p* = .037), but not for the frontal patients (*p* = .124). There was no evidence for a differential semantic interference effect between the controls and the two patient groups (*p*s > .134). Details on the statistics are shown in Table S2. Thus, the pattern of results was the same as reported in the main text.

**Table S2.** Results of the inferential statistics for the response times (RT, top) and error rates (bottom). Results obtained from the full model, unless stated otherwise. Results from the group models are indicated by an asterisk. SE = standard error.

| RT effect | b | SE | t (df) | p |
| --- | --- | --- | --- | --- |
| Congruent vs unrelated | -.096 | .012 | -8.56 (2779) | < .001 |
| Related vs unrelated | .067 | .012 | 7.75 (2780) | < .001 |
| Frontal vs controls | .322 | .079 | 4.09 (24) | < .001 |
| Temporal vs controls | .310 | .067 | 4.61 (25) | < .001 |
| Related vs unrelated: controls* | .068 | .010 | 6.73 (967) | < .001 |
| Related vs unrelated: frontal* | .034 | .022 | 1.54 (318) | .124 |
| Related vs unrelated: temporal* | .037 | .018 | 2.09 (404) | .037 |
| Congruent vs unrelated: frontal vs controls | -.107 | .022 | -4.84 (2781) | < .001 |
| Related vs unrelated: frontal vs controls | -.032 | .023 | -1.42 (2781) | .155 |
| Congruent vs unrelated: temporal vs controls | -.155 | .020 | -7.68 (2783) | < .001 |
| Related vs unrelated: temporal vs controls | -.032 | .021 | -1.36 (2783) | .134 |

Figure S2 shows scatterplots of the relationships between aphasia quotient or lesion volume and the semantic interference effect in response times (RTs) and error rates.


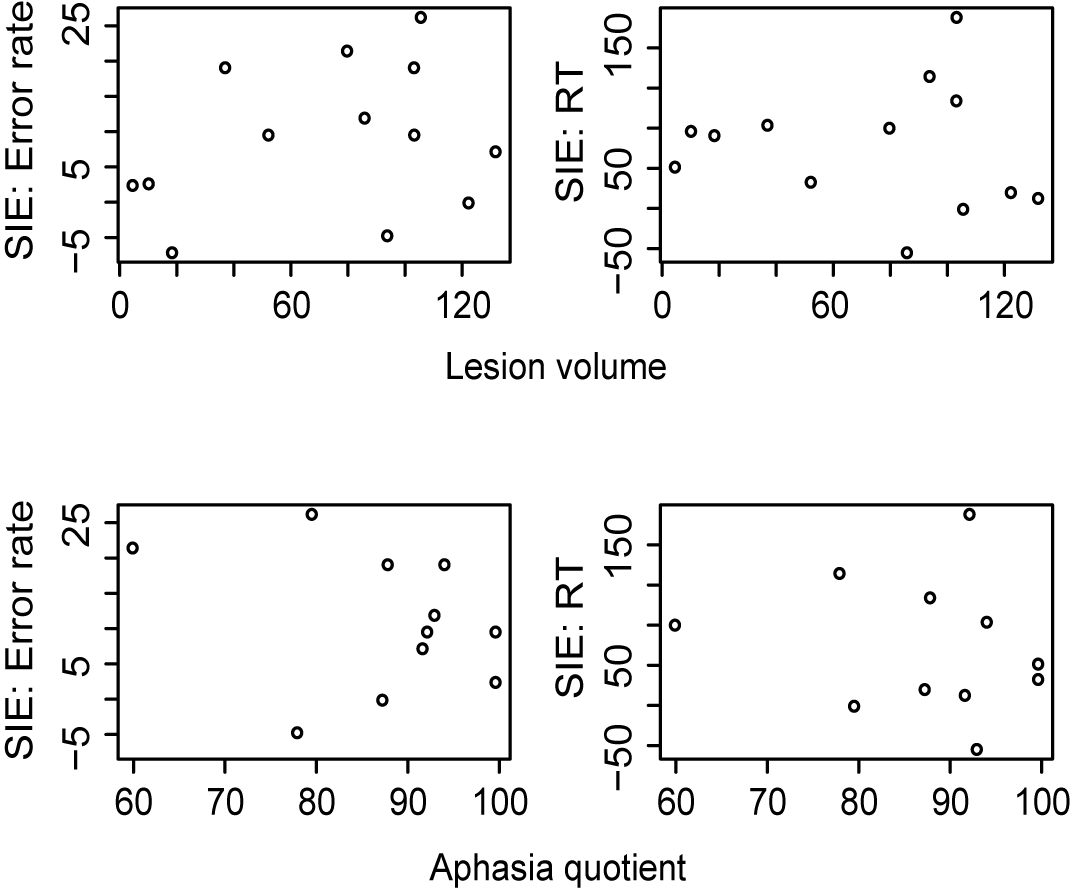


**Figure S2.** Scatterplots for lesion volume (top) and aphasia quotient (bottom) and the semantic interference effect (SIE) in error rates (left) and response times (RT, right).

Lesion volume did not correlate with the magnitude of the semantic interference effect in the RTs (Spearman’s *rho* = -.187, *S* = 432, *p* = .541) nor in the error rates (Spearman’s *rho* = .204, *S* = 290, *p* = .504). Aphasia quotient (available for 11 patients) did not correlate with the magnitude of the semantic interference effect in the RTs (Spearman’s *rho* = -.105, *S* = 243, *p* = .759) nor in the error rates (Spearman’s *rho* = -.149, *S* = 253, *p* = .663).
